# Genomic Insight into Mechanisms of Reversion of Antibiotic Resistance in Multidrug Resistant *Mycobacterium tuberculosis* Induced by a Nanomolecular Iodine-Containing Complex FS-1

**DOI:** 10.3389/fcimb.2017.00151

**Published:** 2017-05-08

**Authors:** Aleksandr I. Ilin, Murat E. Kulmanov, Ilya S. Korotetskiy, Rinat A. Islamov, Gulshara K. Akhmetova, Marina V. Lankina, Oleg N. Reva

**Affiliations:** ^1^Scientific Center for Anti-Infectious DrugsAlmaty, Kazakhstan; ^2^Department of Biochemistry, Centre for Bioinformatics and Computational Biology, University of PretoriaPretoria, South Africa

**Keywords:** *Mycobacterium tuberculosis*, drug resistance reversion, new drug, next generation sequencing, genome polymorphism

## Abstract

Drug induced reversion of antibiotic resistance is a promising way to combat multidrug resistant infections. However, lacking knowledge of mechanisms of drug resistance reversion impedes employing this approach in medicinal therapies. Induction of antibiotic resistance reversion by a new anti-tuberculosis drug FS-1 has been reported. FS-1 was used in this work in combination with standard anti-tuberculosis antibiotics in an experiment on laboratory guinea pigs infected with an extensively drug resistant (XDR) strain *Mycobacterium tuberculosis* SCAID 187.0. During the experimental trial, genetic changes in the population were analyzed by sequencing of *M. tuberculosis* isolates followed by variant calling. In total 11 isolates obtained from different groups of infected animals at different stages of disease development and treatment were sequenced. It was found that despite the selective pressure of antibiotics, FS-1 caused a counter-selection of drug resistant variants that speeded up the recovery of the infected animals from XDR tuberculosis. Drug resistance mutations reported in the genome of the initial strain remained intact in more sensitive isolates obtained in this experiment. Variant calling in the sequenced genomes revealed that the drug resistance reversion could be associated with a general increase in genetic heterogeneity of the population of *M. tuberculosis*. Accumulation of mutations in PpsA and PpsE subunits of phenolpthiocerol polyketide synthase was observed in the isolates treated with FS-1 that may indicate an increase of persisting variants in the population. It was hypothesized that FS-1 caused an active counter-selection of drug resistant variants from the population by aggravating the cumulated fitness cost of the drug resistance mutations. Action of FS-1 on drug resistant bacteria exemplified the theoretically predicted induced synergy mechanism of drug resistance reversion. An experimental model to study the drug resistance reversion phenomenon is hereby introduced.

## Introduction

The dilemma of acquired drug resistance by pathogenic bacteria is generally recognized (Tenover, 2001; Morens et al., 2004). This problem is complicated by a common crisis in development of new antibiotics (Spellberg et al., 2008; van den Boogaard et al., 2009; Piddock, 2012). Drug induced reversion of antibiotic resistance is a prospective approach to combat drug resistant infections. However, the mechanisms of this phenomenon are not understood and even the terminology has not been well defined. In early publications, this term referred to a suppression of antibiotic resistance proteins such as drug efflux pumps and/or beta-lactamases (Reading and Cole, 1977; Rodrigues et al., 2013). Another definition was given in a recent review by Baym et al. (2016), where this phenomenon was considered as an active drug induced counter-selection of resistant variants from populations of pathogens. Several theoretic clauses were discussed in the mentioned paper to explain the resistance reversion that occurred despite the presence of selective antibiotics. Drug induced over-burdening of the resistant strains by an aggravated fitness cost was considered as a possible mechanism. The fitness cost relates to adverse side effects of drug resistance mutations reducing the viability, adaptability, and growth rate of the mutants (Cohen et al., 2003; Gagneux et al., 2006). A mathematic model by Cohen and Murray (2004) has predicted that multidrug resistant pathogens would be rather unlikely to appear due to the cumulated fitness cost. Unfortunately, this prediction was rather over-optimistic. Subsequent compensatory mutations reduce the fitness cost to such a level that the population could retain the mutants for an extended period of time even after the withdrawal of the selective pressure of antibiotics (Pym et al., 2002; Luciani et al., 2009). However, the idea to aggravate the cumulated fitness cost by supplementary drugs in an effort to combat drug resistance still seems attractive.

Tuberculosis remains one of the most dangerous threats to public health. This problem has been complicated by a wide distribution of drug resistant infections that basically brought about a return to the pre-antibiotic era (Cohn et al., 1997; Nouvel et al., 2006; Zager and McNerney, 2008). Multidrug resistant (MDR) tuberculosis pathogens were defined by the WHO as those resistant to isoniazid and rifampicin; and extensively drug resistant (XDR) variants being additionally resistant to fluoroquinalones and at least one second-line injectable antibiotics. Treatment of MDR and XDR tuberculosis requires an application of complex formulations of antibiotics (Iseman, 1993) that often cannot be administered due to life threatening side effects (Awofeso, 2008).

Drug resistance in *Mycobacterium tuberculosis* is associated with spontaneous mutations in functional genomic loci. More than 1,000 putative drug resistance mutations have been predicted (Sandgren et al., 2009). Over the last 40 years, no new antibiotics against tuberculosis have been developed and only recently several new drugs have been proposed: Bedaquiline (Mahajan, 2013), Delamanid (Gupta et al., 2015) and FS-1 (Ilin and Kulmanov, 2014; Kalykova et al., 2016). However, resistance to Bedaquiline and Delamanid has already been reported (Hoffmann et al., 2016). FS-1 is an iodine-containing nanomolecular complex showing an antimicrobial effect (Kalykova et al., 2016). Active units of FS-1 are aggregated micelles containing complexes of triiodide molecules coordinated by metal ions and integrated into a dextrin-polypeptide moiety. The basic formula of the micelle is:

(1) $$\left[\{{\left({\text{L}}_{\text{n}}{\left({\text{MeJ}}_{3}\right)}^{+}\right)}_{\text{y}}{{\left[\text{Me}({\text{L}}_{\text{m}})\text{J}\right]}^{+}}_{\text{x}}\}{\left({\text{Cl}}^{-}\right)}_{\text{y }+\text{ x}+\text{ k}}\right]$$

where L–dextrin-polypeptide ligand; Me–Li/Mg ions; n, m, x, y, and k–variable integers ≥1; molecular mass of the micelles is in the range of 30–300 kD.

In the blood plasma, the micelles bind to blood albumins. The mean residence time (MRT) of FS-1 estimated as a geometric average of the time of elimination of the drug from an organism (Cawello, 1999), was 24.6 h. Disintegration of the micelles causes a dissociation of triiodides into iodine molecules, which are the active antimicrobial agents of FS-1.

FS-1 passed preclinical and clinical trials and in 2015 it was accepted as a new anti-MDR/XDR drug in Kazakhstan (Ilin and Kulmanov, 2014). FS-1 is of great interest for this study because of the reported antibiotic resistance reversion induced by this drug. It was hypothesized that FS-1 could influence the composition of bacterial populations by removal of the most resistant variants of *M. tuberculosis*, probably due to higher sensitivity to oxidative stresses (Cohen and Murray, 2004; Ilin et al., 2017).

The aim of this work was to perform a laboratory experiment on animals infected with a XDR-TB strain to identify genetic changes in bacterial populations induced by FS-1. The strain *M. tuberculosis* SCAID 187.0 was used in this study. It was isolated from a patient with tuberculosis showing extensive drug resistance (Ilin et al., 2015). The present study proved the efficacy of the combinatorial treatment of infected animals by conventional anti-tuberculosis antibiotics supplemented with FS-1. An increased susceptibility to antibiotics was observed in *M. tuberculosis* isolates from the FS-1 treated animals. Sequencing of the isolates demonstrated that FS-1 caused recognizable changes in the *M. tuberculosis* populations by removal of the most resistant clonal lines, which were dominant in the untreated animals and in the animals treated solely by antibiotics. Reduction of the antibiotic resistance correlated with an increased genetic heterogeneity and accumulation of mutated variants of PpsA and truncated PpsE subunits of the phenolpthiocerol polyketide synthase.

## Materials and methods

### Isolation and identification of clinical cultures of *M. tuberculosis*

*M. tuberculosis* strains were isolated on a regular basis from patients' sputum samples during the combinatorial therapy of antibiotics and FS-1 (Ilin et al., 2017). Sputum samples were inoculated into liquid Löwenstein-Jensen medium (HiMedia Laboratories, India) and cultivated at 37°C for 8 weeks. Culture growth was controlled visually, by microscopy of Ziehl–Neelsen stained smears and by standard diagnostic biochemical tests including the positive catalase, niacin and nicotinamidase activities, negative Tween-80 hydrolysis and susceptibility to sodium salicylate (Segal and Bloch, 1956).

### Minimal inhibitory concentration of FS-1

Minimal inhibitory concentration (MIC) of FS-1 was tested on the type strain *M. tuberculosis* H37Rv and several clinical XDR isolates by serial dilution of FS-1 in liquid Löwenstein-Jensen medium with 1.75, 0.35, 0.175, 0.0875, 0.0437, 0.0218, and 0.0109 mg/ml of FS-1. Culture suspensions of 5 × 10^8^ cells/ml were inoculated in aliquots of 0.2 ml into test tubes with 5 ml of the medium with FS-1 and without the compound for the positive growth control. Each concentration of FS-1 was triple replicated. Tubes were incubated for 10 days at 37°C.

### Laboratory animals

In total, 105 healthy male and female 6–8 weeks old Hartley guinea pigs weighing 350–450 g were obtained from the Kazakh Scientific Research Veterinary Institute, Almaty, Kazakhstan. Animals were held under barrier conditions in a biosafety level III animal laboratory at 23 ± 2°C, humidity 50 ± 10%, on a 12 h light/dark cycle. All the animals received the standard forage (Ltd. Assortiment Agro, Russia) and deionized water consumed *ad libitum*.

For blood collection and necropsy, the animals were sacrificed by an overdose of sodium pentobarbital (JSC KievMedPreparat, Ukraine).

The study was approved by the Ethics Committee of the Scientific Center for Anti-Infectious Drugs (no 29, 05.06.2014). Animal care complied with the Guide for the Care and Use of Laboratory Animals (National Research Council, 2010) and with the Kazakhstan governmental guidelines.

### Infection and treatment of the animals by anti-tuberculosis antibiotics and FS-1

Guinea pigs were infected subcutaneously with 1 × 10^6^ CFU/ml of the virulent strain *M. tuberculosis* SCAID 187.0. Bacterial suspensions were prepared *ex tempore* 2 h before infection from the bacterial culture cultivated for 10 days at 37°C on solid Löwenstein-Jensen medium. Development of tuberculosis in the animals was monitored weekly by changes in behavior, general appearance and body weight loss. On the 14th day after infection, three infected animals from the positive control group were sacrificed to confirm the infection state by necropsy and histopathological parameters.

Anti-tuberculosis antibiotics were administrated starting on the 21st day after infection. Antibiotic dosage corresponded to the XDR-TB treatment regimen approved in Kazakhstan by the Ministry of Health. A combined formulation of 4–5 antibiotics is usually applied for XDR-TB treatment (Iseman, 1993). The complex of anti-tuberculosis antibiotics (CAA) included pyrazinamide (*per os* 100 mg/kg), cycloserine (*per os* 20.0 mg/kg), prothionamide (*per os* 20.0 mg/kg), capreomycin (intramuscular 20.0 mg/kg), and amikacin (intramuscular 50.0 mg/kg). FS-1 was administrated *per os* 30 min before CAA in doses of 2.5 and 4.0 mg/kg, which were respectively the therapeutic and maximal tolerable doses of the drug determined during the clinical trials (Kalykova et al., 2016). The treatment course lasted for 60 days. The animals were weighed daily and the dosage of the antibiotics and FS-1 were adjusted individually for every animal. The experimental design is shown in Table 1.

**Table 1:** **The experimental design on laboratory animals (guinea pigs)**.

**No**.	**Group**	**Therapy**	**14th day after infection (control of the infection state)**	**Days of treatment starting from 21st day after infection**							**The recovery group kept under surveillance for 30 days after the experiment**
				**0**	**7**	**14**	**21**	**30**	**45**	**60**	
				**Number of sacrificed animals**							
1	Negative control (uninfected animals)	No treatment	−	−	−	−	−	−	−	3	3
2	Positive control (infected animals)	No treatment	3	3	3	3	3	3	3	3	3
3	Infected animals	CAA	−	3	3	3	3	3	3	3	3
4	Infected animals	CAA+FS-1 (2.5 mg/kg)	−	3	3	3	3	3	3	3	3
5	Infected animals	CAA+FS-1 (4.0 mg/kg)	−	3	3	3	3	3	3	3	3

### Blood sampling and histological studies

Blood and tissue collections were performed for hematological, biochemical, microbiological and histological studies on the 14th and 21st days after the infection and on the 14th, 30th, 45th and 60th days of the treatment. Whole blood collection was performed by a cardiac puncture of the narcotized animals. Blood samples were placed in 1 ml Vacumed test tubes with K3-EDTA anticoagulant (FL Medical, Italy) and then analyzed by the Analyzer Human Humacount (Human GmbH, Germany). The remaining volumes of the blood samples were placed in 1.5 ml vials without anticoagulants and allowed to clot at room temperature for 60 min. The samples were then centrifuged at 1800 × g for 5 min. The serum was decanted and stored at −80°C prior to the analysis by Analyzer A25 (BioSystems Diagnostics Pvt. Ltd., India) to identify concentrations of the chemical compounds. The statistical reliability of observed differences was confirmed by Student's *t*-test. *P*-values smaller or equal to 0.05 were considered as significant.

### Necropsy

Pieces of 10 × 20 mm were sampled in several repeats from the areas in-between necrolysis and intact tissues from lungs, livers, spleens, kidneys and thyroid glands of the sacrificed animals. The samples were fixed in 10% paraformaldehyde (Sigma, USA) buffered with PBS (Sigma, USA) at pH 7.0. Paraffin embedding and sectioning were performed by using the conventional methodology (Ross and Pawlina, 2006). Then the tissue samples were trimmed and processed by microscopic examination after staining with hematoxylin and eosin (Sigma, USA), Masson's trichrome and Van Gieson's stains (Bio Optica, Italy).

### Isolation and storage of *M. tuberculosis* cultures from the infected animals

Tissue samples of approximately the same size (10 × 20 mm) were homogenized in sterilized laboratory mortars and re-suspended in 4 volumes of 6% sulphuric acid by shaking for 10 min at room temperature. The obtained suspension was left to precipitate and the supernatant fluid was removed. Deposited materials were washed out 3 times with 4 volumes of sterile saline (0.9% NaCl in distilled water) and the aliquots of 1 ml were inoculated onto solid Löwenstein-Jensen medium for further cultivation at 37°C for 8 weeks. Culture growth was controlled visually. Selected colonies (from 10 to 25 per group of isolates) were checked individually for susceptibility to antibiotics. The isolated cultures were frozen at −80°C in a protective medium (3% gelatin and 10% sucrose in water) and then lyophilized by ALPHA 1-2 LD-plus (Martin Christ Gefriertrocknungsanlagen GmbH, Germany) for extended storage at −30°C.

### Susceptibility to antibiotics

Susceptibility to all antibiotics but pyrazinamide was tested by growing the cultures for 4 weeks at 37°C in test-tubes on solid Löwenstein-Jensen medium supplemented with antibiotics in recommended concentrations (Krüüner et al., 2006). Medium pH was adjusted to 6.8. Susceptibility to pyrazinamide was tested on solid medium FAST-3L (BIOK, Russia) with pH adjusted to 5.0. The following antibiotics in μg/ml were used: isoniazid (0.2), rifampicin (40.0), streptomycin (4.0), pyrazinamide (200.0), ethambutol (2.0), amikacin (30.0), kanamycin (30.0), capreomycin (40.0), ofloxacin (2.0), cycloserine (40.0), and ethionamide (40.0). Percentages of drug resistant variants among *M. tuberculosis* isolates from different groups of infected animals were calculated. To compare the percentages of drug resistance in different groups, the standard error was calculated as the square root of [*p* × (1–*p*)] / *n*, where *p*, an average percentage of drug resistant isolates in a group; *n*, number of repeats of the experiment.

Minimal inhibition concentrations (MIC) of the antibiotics were determined by the serial dilution method in a synthetic nutrient medium containing: KH_2_PO_4_—1.5 g/l, Na_2_HPO_4_—2.5 g/l, MgSO_4_× 7H_2_O—0.5 g/l, sodium citrate—1.5 g/l, ammonium iron (III) hydrocitrate—0.05 g/l, L-asparagine—0.1 g/l, glycerol—30 ml, sucrose—220 g/l, bovine serum (Sigma Aldrich)—100 ml/l and bacteriological agar (HiMedia Laboratories, India)—0.5 g/l. Antibiotics were dissolved in the medium to obtain the required serial dilutions. Then the diluted solutions of the antibiotics were dispensed by 700 μl per well into 48-well plates (BD Falcon, NU, USA). Each well was inoculated with 100 μl of *ex tempore* prepared suspensions of bacterial cells, 10^8^ CFU/ml. Plates were incubated for 8 days at 37°C. Then aliquots of 100 μl of 0.05% (w/v) resazurin (Sigma-Aldrich, St. Louis, MO) were added to each well and the plates were incubated for a further 24 h. Bacterial growth was detected by changes in the medium color from violet to pink. MIC values were identified in 3 repeats. Medium contamination with and without antibiotics and the growth of *M. tuberculosis* on the medium without antibiotics were controlled. The following serial dilutions of the antibiotics were used: isoniazid—0.35, 0.3, 0.25, 0.2, 0.15, 0.1, and 0.05 μg/ml; rifampicin—6.0, 5.0, 4.0, 3.0, 2.0, 1.0, and 0.5 μg/ml; streptomycin—6.0, 5.0, 4.0, 3.0, 2.0, 1.0, and 0.5 μg/ml; pyrazinamide—350, 300, 250, 200, 150, 100, and 50 μg/ml; and ethionamide—17.5, 15.0, 12.5, 10.0, 7.5, 5.0, and 2.5 μg/ml.

### DNA extraction, sequencing and comparison

DNA samples were extracted from the isolates by the Cetyltrimethylammonium bromide (CTAB) method (Murray and Thompson, 1980). The eluted DNA was quantified using the Qubit dsDNA BR Assay Kit (Life Technologies, USA). In total, twelve DNA samples were prepared (Table 2) and sequenced by Macrogen (South Korea) using Illumina Hiseq 2000 paired-end technology.

**Table 2:** **Sequencing of ***M. tuberculosis*** isolates from the infected animals on different days of the experiment**.

**Experimental group**	**Day of isolation after beginning of the treatment and the sample codes**	**Number of generated paired-end DNA reads**	**EMBL-EBI sample references**
Clinical isolate of *M. tuberculosis* SCAID 187.0 used for infection of the animals		36,366,100	SAMEA4079245
Group 2, positive control (infected animals)	30th day, sample 2_30_16	10,546,455	SAMEA4079234
	45th day, sample 2_45_7	25,646,205	SAMEA4079236
	45th day, sample 2_45_30	10,362,885	SAMEA4079235
Group 3, animals treated with CAA	30th day, sample 3_30_46	27,488,724	SAMEA4079237
	60th day, sample 3_60_51	10,290,171	SAMEA4079238
Group 4, animals treated with CAA+FS-1 (2.5 mg/kg)	30th day, sample 4_30_75	10,463,777	SAMEA4079239
	60th day, sample 4_60_76	20,571,859	SAMEA4079240
	60th day, sample 4_60_77	10,091,644	SAMEA4079241
Group 5, animals treated with CAA+FS-1 (4.0 mg/kg)	30th day, sample 5_30_104	9,987,928	SAMEA4079242
	60th day, sample 5_60_81.1	10,537,399	SAMEA4079243
	60th day, sample 5_60_81.2	19,905,283	SAMEA4079244

Variant calling was performed by aligning the DNA reads to the reference sequence SCAID 187.0 [CP012506] using CLC Genomics Workbench 7.0.3. Statistical validation of the identified variants was performed by the Quality based variant detection algorithm with the PHRED-type quality cut-off set to 20 for the central nucleotide residue and to 15 for five neighboring residues. Maximum of two gaps or mismatches were allowed per read.

## Results

The experiment on laboratory animals was designed to simulate the clinical trial of FS-1 and induction of an antibiotic resistance reversion under the selective pressure of antibiotics. MIC of FS-1 was identified for the clinical isolate *M. tuberculosis* SCAID 187 during the first isolation and after 60 days of the combinatorial treatment by CAA+FS-1. Prior to the treatment, the determined MIC was 27.7 ± 2.4 μg/ml and after the treatment course the isolates from the same patients became even more sensitive to FS-1 with the MIC values 21.1 ± 2.0 μg/ml. This indicates that the application of FS-1 did not cause any resistance selection for the drug. An interesting observation was that the clinical XDR-TB isolate was even more sensitive to FS-1 than the drug sensitive type strain *M. tuberculosis* H37Rv (MIC was around 48 μg/ml). However, the hypothesis of a higher susceptibility of multidrug resistant variants has yet to be confirmed in an additional study with a greater number of isolates.

### Drug resistance reversion induced *in vitro* by FS-1

It was found that the cultivation of the XDR strain SCAID 187.0 for 60 days in six passages on the medium with a sub-lethal dose of 12 μg/ml of FS-1 reversed the susceptibility of the bacteria to the tested antibiotics. MIC values determined for the cultures cultivated with FS-1 compared to the control condition (cultivated on the same medium without FS-1) are shown in Table 3 (see also Supplementary Figure 1). The values in Table 3 were confirmed in three replications of the experiment. More than two folds MIC reduction observed for rifampicin, ethionamide and pyrazinamide was considered as statistically reliable. These results cannot be attributed to any direct interaction between FS-1 and the antibiotics as the susceptibility was tested on the medium without FS-1. The increased susceptibility to the antibiotics may be explained by a counter-selection of the most resistant variants from the population.

**Table 3:** **Drug resistance reversion induced ***in vitro*** by FS-1 in the XDR strain SCAID 187.0**.

**Antibiotic**	**MIC (μg/ml) after 60 days of cultivation without FS-1**	**MIC (μg/ml) after 60 days of cultivation with 12 μg/ml of FS-1**
Isoniazid	0.25 ± 0.05	0.2 ± 0.05
Rifampicin	5.0 ± 1.0	1.0 ± 0.5
Streptomycin	6.0 ± 1.0	4.0 ± 1.0
Pyrazinamide	250 ± 50.0	100 ± 50.0
Ethionamide	12.5 ± 2.5	5 ± 2.5

### Combinatorial treatment of XDR-TB infected laboratory animals with antibiotics and FS-1

**Figure 1:**
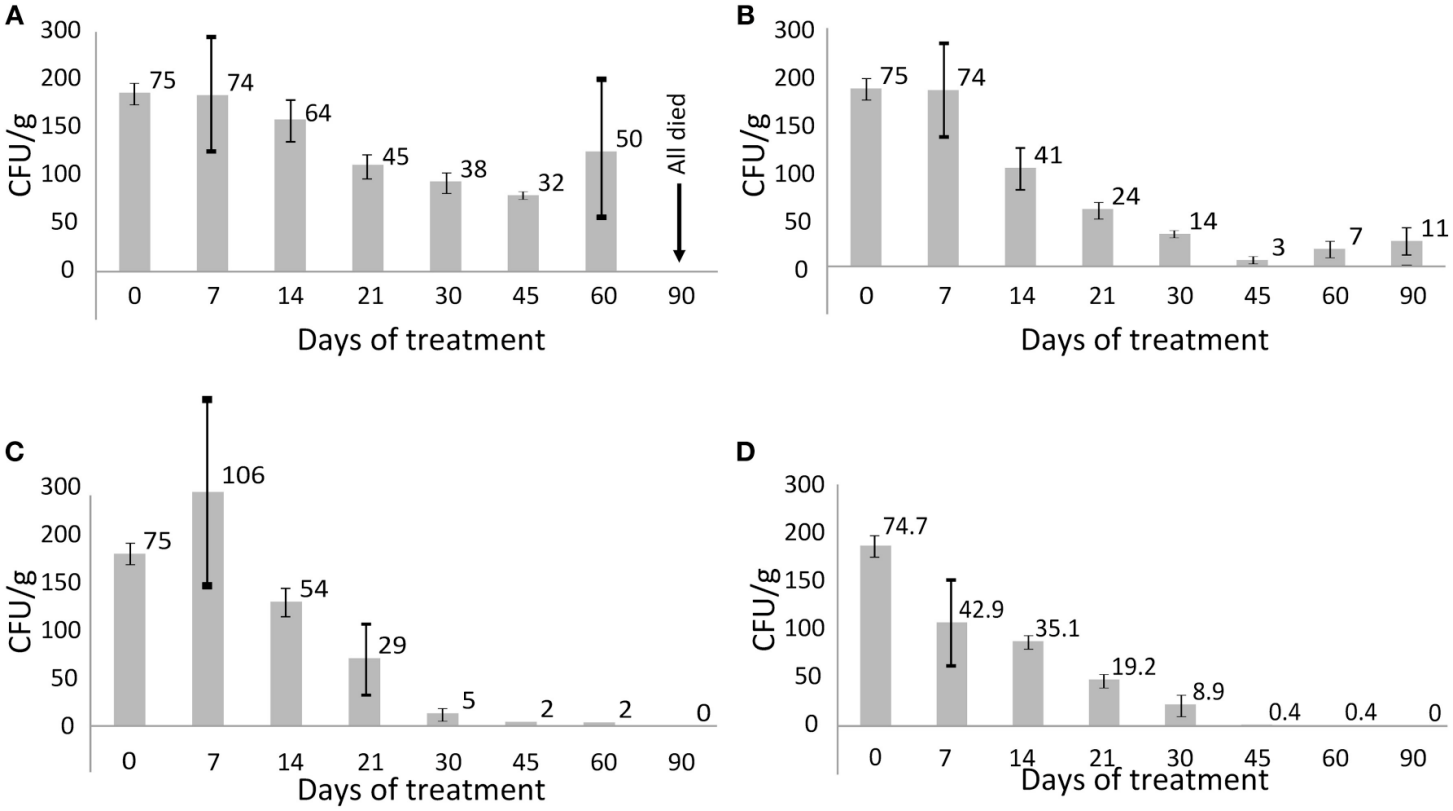
**CFU titres in different groups of animals infected with the XDR ***M. tuberculosis*****. Average values are indicated and the bars depict standard deviations. **(A)** Group 2, positive control (untreated animals); **(B)** group 3 treated with CAA; **(C)** group 4 treated with CAA+FS-1 (2.5 mg/kg); **(D)** group 5 treated with CAA+FS-1 (4.0 mg/kg).

Miliary tuberculosis was diagnosed by necropsy of the sacrificed animals and by microscopy of histological sections. Granulomatous lungs, spleens and livers with multiple disseminated tubercles and significantly enlarged lymph nodes were observed (Supplementary Figure 2). Untreated animals died during the 60 days of the experiment. Contrary, the infected animals subjected to combinatorial treatment with FS-1 in doses of 2.5 and 4 mg/kg recovered from the disease. Fast recovery of these animals could even be seen by the positive body weight dynamics compared to the other groups of animals (Supplementary Table 1). The antibiotic therapy without FS-1 showed only a transient effect followed by a subsequent recurrence of the disease during the 30 days of the recovery period (Figure 1). Necropsy of the sacrificed animals and the results of the biochemical blood tests also confirmed the positive effect of FS-1 (Supplementary Figure 3 and Supplementary Table 2). In general, the biochemical blood test parameters in the groups treated by CAA+FS-1 corresponded to those in the intact animals of the negative control group (Supplementary Table 2). It was concluded that the hepatotoxicity of the anti-tuberculosis antibiotics was alleviated by FS-1 either by a direct action on hepatocytes, or by a faster removal of the tuberculosis infection. Differences revealed by these tests between the animals administrated with 2.5 and 4.0 mg/kg of FS-1 were statistically insignificant.

*M. tuberculosis* isolates from the animals treated for 45 days by CAA+FS-1 showed an increased susceptibility to the antibiotics compared to the isolates from the positive control group and especially to those treated solely by CAA (Table 4). There was a clear trend of a dose dependent drug resistance reversion induced by FS-1 despite some variations between the isolates from different animals. Contrary, the treatment of the animals infected with XDR-TB solely by CAA caused a selection of more resistant variants.

**Table 4:** **Percentage ranges of drug resistance among the isolates from the infected animals**.

**Antibiotics**	**Stock culture (%)**	**Group 2, positive control (%)**	**Group 3, CAA (%)**	**Group 4, CAA+FS-1 (2.5 mg/kg) (%)**	**Group 5, CAA+FS-1 (4.0 mg/kg) (%)**
Isoniazid	100	100	100	75.0–83.3	75.0–80.0
Rifampicin	100	100	100	83.3–87.5	^*^50.0–60.0
Streptomycin	100	100	100	100	80.0–75.0
Pyrazinamide	80.0–92.0	83.3	100	^*^33.3–37.5	^*^20.0–25.0
Ethambutol	88.0–100	83.3	100	66.7–75.0	75.0–80.0
Amikacin	0.0–4.0	0.0	^*^16.7–20.0	0.0	0.0
Kanamycin	60.0–80.0	66.7–83.3	83.3–90.0	50.0	^*^20.0–25.0
Capreomycin	48.0–60.0	33.3–66.7	80.0–83.3	62.5–66.7	50.0–60.0
Ofloxacin	56.0–60.0	66.7	33.3	0.0–12.5	50.0–60.0
Cycloserine	16.0–20.0	33.3	66.7–70.0	0.0–25.0	0.0
Ethionamide	60.0–72.0	50.0	83.3–90.0	0.0–12.5	^*^0.0

*Every condition was repeated in triplicate and the minimal and maximal percentages of drug resistant isolates (if different) are shown*.

* *The difference in the percentage of drug resistant variants in this group compared to the positive control group 2 is statistically reliable with p-value ≤ 0.05*.

### Comparison of genome sequences of *M. tuberculosis* isolates

Eight drug resistance mutations listed in Table 5 were found in the reference genome *M. tuberculosis* SCAID 187.0 (CP012506). All of them remained unchanged in the isolates showing reduced drug resistance (Table 4). This finding was consistent with the results of sequencing of a series of clinical isolates obtained during the clinical trial of FS-1 (Ilin et al., 2017). Variant calling in the experimental isolates revealed 30 polymorphic sites, which were found in at least two isolates per group with the frequency of minor alleles above 1% and a read coverage above 10 (Table 6). Only two polymorphic loci were present in the isolates from the animals treated solely by CAA. This indicates a purifying selection caused by the antibiotics. Fifteen polymorphic loci were discovered in the isolates from the untreated animals. *M. tuberculosis* isolates from the animals treated by CAA+FS-1 showed the highest genetic heterogeneity with 20 and 26 identified polymorphic loci in groups 4 and 5, respectively. Almost all polymorphic loci were in hyper-variable PPE-PGRS genes. However, the treatment of the animals with FS-1 caused an accumulation of mutations in subunits A and E of the phenolpthiocerol synthase. One mutation causing a P-to-H substitution in a proline rich linker region between the acyltransferase and dehydratase domains was characteristic for 37–38% of the DNA reads generated from the isolates of groups 4 and 5. A frameshift mutation caused by an insertion of 2 nucleotides in the mid part of the *ppsE* subunit was found in 95% of the reads generated from the isolates on the 30th day of the treatment by CAA + 4.0 mg/kg FS-1. In the *M. tuberculosis* cultures isolated from animals in this group on the 60th day of the treatment the percentage of this mutation lowered to 50%. These mutations in *ppsA* and *ppsE* were not found in the initial strain SCAID 187.0 or in the isolates from the other groups of animals.

**Table 5:** **Canonical drug resistance mutations found in the genome of the strain ***M. tuberculosis*** SCAID 187.0 [CP012506]**.

**Gene**	**Location in CP012506**	**Codon**	**DNA locus**	Amino acid substitution^*^	**Associated resistance**
Rv0006, *gyrA*	7,585	95	AGC/ACC	Ser→Thr	Fluoroquinolones (Kapur et al., 1999)
Rv0682, *rpsL*	781,687	43	AAG/AGG	Lys→Arg	Streptomycin (Finken et al., 1993)
Rv1908c, *katG*	2,155,168	315	AGC/ACC	Ser→Thr	Putative isoniazid resistance
Rv1908c, *katG*	2,154,724	463	CGG/CTG	Arg→Leu	Isoniazid (Heym et al., 1995)
Rv2043c, *pncA*	2,288,747	141	CAG/CCG	Gln→Pro	Pyrazinamide (Scorpio and Zhang, 1996)
Rv2247, *accD6*	2,521,428	229	GAC/GGC	Asp→Gly	Isoniazid (Ramaswamy et al., 2003)
Rv3795, *embB*	4,247,431	306	TGG/TTG	Met→Ile	Ethambutol (Sreevatsan et al., 1997)
Rv3919, *gidB*	4,407,927	92	ACC/AAC	Glu→Asp	Streptomycin (Okamoto et al., 2007)

* *In pairs of amino acid substitutions the residue to the left is the state in the type strain H37Rv (drug sensitive) and the residue to the right is the state in the MDR strain SCAID 187.0*.

**Table 6:** **Genomic polymorphisms in ***M. tuberculosis*** isolates from different groups of experimental animals identified by the quality based variant calling against the reference genome SCAID 187.0 [CP012506]**.

**Locus**	**Annotation**	Location^*^	**Type of polymorphism^†^**	**Nucleotide substitution**	**Percentage of mutations in different groups of Mtb isolates**			
					**Group 2 (positive control)**	**Group 3 (CAA)**	**Group 4 (CAA+2.5 mg/kg FS-1)**	**Group 5 (CAA+4.0 mg/kg FS-1)**
AFL40_0134	Type V PE-PGRS2 protein; similar to Rv0124	148907	SNV G/D	G/A			96.67 ± 3.33	
AFL40_0295	Type V PE-PGRS3 protein; similar to Rv0278c; involved in virulence, detoxification, adaptation	332900	SNV A/T	C/T	100		100	100
		334374	SNV	C/T	100			
AFL40_0296	Type V PE-PGRS4 protein; similar to Rv0279c	336824	SNV	A/G	62.51 ± 3.69		56.11 ± 5.08	59.79 ± 1.46
		336827	SNV	C/G	61.68 ± 1.48		56.69 ± 6.69	57.28 ± 0.94
		336845	SNV	C/G			67.4 ± 4.89	70.57 ± 1.16
AFL40_1822	Type V PPE24 protein; similar to Rv1753c	1972684	SNV P/A	G/C	100		100	100
		1972687	SNV P/A	G/C	100		100	100
AFL40_1946	Hypothetical protein; similar to Rv1883c	2116184	SNV V/A	A/G	55.63 ± 0.66	62.18 ± 1.1	51.07 ± 7.17	58.80 ± 2.37
AFL40_1980a	Putative PPE family protein	2146279	SNV N/D	T/C	39.80 ± 1.93	39.84 ± 0.03	40.57 ± 1.91	46.59 ± 2.07
Non-coding	Possible promoter of peptidase Rv2141c	2397748	SNV	T/C	38.62 ± 0.23			37.45 ± 0.09
AFL40_2592	Type V PE_PGRS43 protein; similar to Rv2490c	2800107	SNV	A/C			44.89 ± 8.05	
AFL40_3034	Phenolpthiocerol synthesis type-I polyketide synthase; similar to Rv2931	3237781	SNV P/H	C/A			37.59 ± 1.88	38.66 ± 1.88
AFL40_3038	Phenolpthiocerol synthesis type-I polyketide synthase PpsE; similar to Rv2935	3259526–3259527	INS frameshift	del/GT				63.71 ± 22.1
AFL40_3247	Type IV PPE51 protein; similar to Rv3136; involved in cell wall processes	3494584	SNV	G/A	99.71 ± 0.41		100	100
AFL40_3271	Putative PPE family protein	3521259–3521261	DEL A/del	CGC/del	66.56 ± 6.23		63.91 ± 4.84	62.19 ± 2.46
Non-coding	Spacer region between Rv3401 and Rv3402c	3821160	SNV	G/A	81.12 ± 1.95		83.54 ± 1.71	83.12 ± 1.92
Non-coding	Spacer region between Rv3401 and Rv3402c	3821198–3821199	DEL	CA/del			36.4 ± 1.1	
Non-coding	Spacer region between Rv3401 and Rv3402c	3821241	SNV	A/G	79.37 ± 1.97		80.48 ± 1.5	84.87 ± 0.56
AFL40_3563	hypothetical protein	3849170	SNV	C/T	63.20 ± 1.73		54.89 ± 4.89	58.89 ± 2.89
AFL40_3645	Type V PE_PGRS53 protein; similar to Rv3507	3930499	SNV	C/T			100	100
AFL40_3651	Type V PE_PGRS55 protein; similar to Rv3511	3944852	SNV	C/T			92.5 ± 7.5	100
		3944859–3944860	MNV T/D	AC/GA				83.33 ± 16.6
		3944865–3944866	INS frameshift	del/T				49.26 ± 3.11
		3944872	DEL frameshift or together with the previous INS: AR/VG	C/del				49.39 ± 3.94
		3944866	SNV G/A	G/C				47.02 ± 5.35
		3944875	SNV V/A	G/C				41.42 ± 0.24
		3944878	SNV D/G	A/G				56.06 ± 10.6
		3944885	DEL frameshift	A/del				44.84 ± 8.48
		3944888–3944889	INS frameshift or together with the previous DEL: Q/N	del/T				39.23 ± 2.87
AFL40_3653	Type V PE_PGRS61 protein; similar to Rv3508	3949371–3949372	MNV G/A	GG/CA	68.48 ± 18.4		80.16 ± 12.1	
Number of polymorphisms per genome:					15	2	20	26

* Locations are given as in the reference genome SCAID 187.0 [CP012506];

† *Types of polymorphisms are: SNV, single nucleotide polymorphism; MNV, multiple nucleotide polymorphism; INS, insertion; DEL, deletion. In the case of sense nucleotide substitutions, the corresponding amino acid substitutions are shown*.

## Discussion

The experiment on laboratory animals confirmed the therapeutic effect of FS-1 administrated in combination with the antibiotics of the standard regiment. It was hypothesized that the induced drug resistance reversion presumably contributed to the therapeutic effect by FS-1. Remarkably, the drug resistance reversion was demonstrated also *in vitro* where no direct interaction between FS-1 and the antibiotics was possible. There are evidences that FS-1 can cause a counter-selection of the drug resistant variants of *M. tuberculosis* (Ilin et al., 2017).

Sequencing of the isolates obtained from different groups of animals revealed dissimilar frequencies and allelic states of genomic polymorphisms. Selection of the resistant clonal lines was caused by the CAA treatment. Contrary, no resistance selection was observed in the *M. tuberculosis* isolates from the animals threated by CAA+FS-1. The combinatorial therapy led to a complete sanitation of the infected animals from Mycobacterial bacilli in 45–60 days of the treatment (Figure 1) which was also confirmed by the blood test results and necropsy (Supplementary Figures 2–3, Supplementary Tables 1–2).

*M. tuberculosis* isolates obtained during the combinatorial treatment showed an increased susceptibility to antibiotics (Table 4) associated with an increased level of genetic heterogeneity (Table 6). However, all the “canonical” drug resistance mutations identified in the initial strain *M. tuberculosis* SCAID 187.0 (Table 5) remained intact in the isolates despite the increased susceptibility. This suggests that the known antibiotic resistance mutations might not be sufficient to produce sustainable drug resistance which requires the presence of a specific genetic context rendered by other polymorphic genes.

Majority of the polymorphic loci were located in hyper-variable PPE-PGRS genes. These genes are three fold more mutable than the average. It was hypothesized that their major role was an antigenic redress of bacterial cells (Banu et al., 2002). Several additional functions of PPE-PGRS proteins were proposed in a recent publication by Fishbein et al. (2015). Selection of PPE-PGRS mutants was also observed in *M. tuberculosis* cultures grown in human macrophages (Guerrini et al., 2016). However, it should also be considered that these mutations could just be genetic markers of different competing clonal lines in the population, which differ in gene transcription or DNA methylation patterns (Heusipp et al., 2007).

CAA+FS-1 treatment of the infected animals caused an accumulation of disruptive mutations in *ppsE* and *ppsA*. A similar result was obtained recently by sequencing a series of TB isolates from patients treated with FS-1 during clinical trials (Ilin et al., 2017). This polyketide synthase operon encodes synthesis of a methyl-branched fatty acid phthiocerol dimycocerosate (PDIM) that is a characteristic cell wall compound of Mycobacteria (Azad et al., 1997; Camacho et al., 2001). PDIM has been proven to be an important virulence factor (Yu et al., 2012). However, other authors noted that impairing of the PDIM biosynthesis improved the persistence ability of the pathogens (Torrey et al., 2016). Variations in the PDIM operon may allow finding a compromise between pathogenicity, viability and ability to persist. An interesting observation was that the frequency of the frameshift mutations in *ppsE* reached the 95% maximum after 30 days of the treatment by CAA + 4 mg/kg FS-1, but then decreased to 50% implying that the persistent variants did not overcome the continuous treatment by FS-1.

The observed increase in the genetic heterogeneity of the isolates under influence of FS-1 may indicate an accumulation of random mutations causing a general metabolic retardation. Indeed, on average it took longer to mature the isolates on the late stages of the treatment with FS-1; however, the growth rate results were inconsistent. Such strains could persist for longer in the treated animals without being able to maintain the infection, while in the animals treated solely by CAA the selected drug resistant variants caused recurrences of the disease (Figure 1). Counter-selection of the drug resistant variants from the population may be associated with the fitness cost aggravation by FS-1 (Ilin et al., 2017). It was reported that the drug resistant mutants were more sensitive to oxidative stress (Cohen and Murray, 2004). Under the combinatorial action of the antibiotics and FS-1, the XDR-TB population failed to find genetic variants fitting both selective pressures. Late isolates appeared to be more susceptible to the antibiotics and to FS-1. To the best of our knowledge, this work was the first experimental model to study the drug induced antibiotic resistance reversion by the induced synergy mechanism that had been predicted theoretically in a recent publication (Baym et al., 2016). Application of drug resistance reversion induced in clinics may allow reducing the frequency of side effect complications by shortening the treatment course of the standard antibiotics and it will improve the efficacy of curing of XDR-TB infection that currently is below a 20% success rate (Pietersen et al., 2014).

## Author contributions

AI and MK planning of experiments and general managements; RI, GA, and ML work with *M. tuberculosis* isolates, biochemical experiments; RI work with laboratory animals; IK extraction of DNA and sequencing; OR bioinformatics support; AI, RI, ML, and OR preparation and edition of the manuscript.

## Funding

The research was funded by the grant 0115PK00389 provided by the Ministry for investments and development of Kazakhstan. Bioinformatic analysis was funded by the grant #93664 provided for ONR by the National Research Foundation (NRF) of South Africa.

### Conflict of interest statement

The authors declare that the research was conducted in the absence of any commercial or financial relationships that could be construed as a potential conflict of interest.

## Abbreviations

MDR-TB: multidrug resistant tuberculosis
CAA: complex of anti-tuberculosis antibiotics
PDIM: phthiocerol dimycocerosate
SCAID: Scientific Center for Anti-Infectious Drugs
XDR-TB: extensively drug resistant tuberculosis.

## References

[B1] Awofeso N. (2008). Anti-tuberculosis medication side-effects constitute major factor for poor adherence to tuberculosis treatment. Bull. World Health Organ. 86, B–D. 10.2471/blt.07.043802 18368191 PMC2647396

[B2] Azad A. K. Sirakova T. D. Fernandes N. D. Kolattukudy P. E. (1997). Gene knockout reveals a novel gene cluster for the synthesis of a class of cell wall lipids unique to pathogenic mycobacteria. J. Biol. Chem. 272, 16741–16745. 10.1074/jbc.272.27.16741 9201977

[B3] Banu S. Honore N. Saint-Joanis B. Philpott D. Prevost M. C. Cole S. T. (2002). Are the PE-PGRS proteins of *Mycobacterium tuberculosis* variable surface antigens? Mol. Microbiol. 44, 9–19. 10.1046/j.1365-2958.2002.02813.x 11967065

[B4] Baym M. Stone L. K. Kishony R. (2016). Multidrug evolutionary strategy to reverse antibiotic resistance. Science 351:aad3292. 10.1126/science.aad3292 26722002 PMC5496981

[B5] Camacho L. R. Constant P. Raynaud C. Laneelle M. A. Triccas J. A. Gicquel B.. (2001). Analysis of the phthiocerol dimycocerosate locus of *Mycobacterium tuberculosis*. Evidence that this lipid is involved in the cell wall permeability barrier. J. Biol. Chem. 276, 19845–19854. 10.1074/jbc.M100662200 11279114

[B6] Cawello W. (1999). Parameters for Compartment Free Pharmacokinetics: Standardisation of Study Design, Data Analysis and Reporting. Aachen: Shaker Verlag.

[B7] Cohen T. Murray M. (2004). Modeling epidemics of multidrug-resistant *M. tuberculosis* of heterogeneous fitness. Nat. Med. 10, 1117–1121. 10.1038/nm1110 15378056 PMC2652755

[B8] Cohen T. Sommers B. Murray M. (2003). The effect of drug resistance on the fitness of *Mycobacterium tuberculosis*. Lancet Infect. Dis. 3, 13–21. 10.1016/S1473-3099(03)00483-3 12505028

[B9] Cohn D. L. Bustreo F. Raviglione M. C. (1997). Drug-resistant tuberculosis: review of the worldwide situation and the WHO/IUATLD Global Surveillance Project. Clin. Infect. Dis. 24, S121–S130. 10.1093/clinids/24.Supplement_1.S121 8994791

[B10] Finken M. Kirschner P. Meier A. Wrede A. Böttger E. C. (1993). Molecular basis of streptomycin resistance in *Mycobacterium tuberculosis*: alterations of the ribosomal protein S12 gene and point mutations within a functional 16S ribosomal RNA pseudoknot. Mol. Microbiol. 9, 1239–1246. 10.1111/j.1365-2958.1993.tb01253.x 7934937

[B11] Fishbein S. van Wyk N. Warren R. M. Sampson S. L. (2015). Phylogeny to function: PE/PPE protein evolution and impact on *Mycobacterium tuberculosis* pathogenicity. Mol. Microbiol. 96, 901–916. 10.1111/mmi.12981 25727695

[B12] Gagneux S. Long C. D. Small P. M. Van T. Schoolnik G. K. Bohannan B. J. (2006). The competitive cost of antibiotic resistance in *Mycobacterium tuberculosis*. Science 312, 1944–1946. 10.1126/science.1124410 16809538

[B13] Guerrini V. Subbian S. Santucci P. Canaan S. Gennaro M. L. Pozzi G. (2016). Experimental evolution of *Mycobacterium tuberculosis* in human macrophages results in low-frequency mutations not associated with selective advantage. PLoS ONE 11:e0167989. 10.1371/journal.pone.0167989 27959952 PMC5154527

[B14] Gupta R. Geiter L. J. Wells C. D. Gao M. Cirule A. Xiao H. (2015). Delamanid for extensively drug-resistant tuberculosis. N. Engl. J. Med. 373, 291–292. 10.1056/NEJMc1415332 26176402

[B15] Heusipp G. Fälker S. Schmidt M. A. (2007). DNA adenine methylation and bacterial pathogenesis. Int. J. Med. Microbiol. 297, 1–7. 10.1016/j.ijmm.2006.10.002 17126598

[B16] Heym B. Alzari P. M. Honoré N. Cole S. T. (1995). Missense mutations in the catalase-peroxidase gene, *katG*, are associated with isoniazid resistance in *Mycobacterium tuberculosis*. Mol. Microbiol. 15, 235–245. 10.1111/j.1365-2958.1995.tb02238.x 7746145

[B17] Hoffmann H. Kohl T. A. Hofmann-Thiel S. Merker M. Beckert P. Jaton K.. (2016). Delamanid and Bedaquiline resistance in *Mycobacterium tuberculosis* ancestral Beijing genotype causing extensively drug-resistant tuberculosis in a Tibetan Refugee. Am. J. Respir. Crit. Care Med. 193, 337–340. 10.1164/rccm.201502-0372LE 26829425

[B18] Ilin A. I. Kulmanov M. E. (2014). Antibacterial Agent for Treating Infectious Diseases of Bacterial Origin. Patent US20140010782 A1—2014-09-03. Available online at: https://www.google.com/patents/US20140010782

[B19] Ilin A. I. Kulmanov M. E. Korotetskiy I. S. Akhmetova G. K. Lankina M. V. Shvidko S. V.. (2015). Complete genome sequence of the multidrug- resistant clinical isolate *Mycobacterium tuberculosis* 187.0, used to study an effect of drug susceptibility reversion by a new medicinal drug FS-1. Genome Announc. 3:e01272-15. 10.1128/genomeA.01272-15 26543112 PMC4645197

[B20] Ilin A. I. Kulmanov M. E. Korotetskiy I. S. Lankina M. V. Akhmetova G. K. Shvidko S. V. (2017). Constraints of drug resistance in *Mycobacterium tuberculosis*–prospects for pharmacological reversion of susceptibility to antibiotics. Open Conf. Proc. J. 8, 33–43. 10.2174/2210289201708010033

[B21] Iseman M. D. (1993). Treatment of multidrug-resistant tuberculosis. N. Engl. J. Med. 329, 784–791. 10.1056/NEJM199309093291108 8350889

[B22] Kalykova A. Kustova T. Sakipova Z. Ibragimova N. Islamov R. Vetchý D. (2016). Acute and subchronic toxicity studies of the original drug FS-1. Acta Vet. Brno 85, 9–16. 10.2754/avb201685010009

[B23] Kapur V. Li L. L. Hamrick M. R. Plikaytis B. B. Shinnick T. M. Telenti A.. (1999). Rapid *Mycobacterium* species assignment and unambiguous identification of mutations associated with antimicrobial resistance in *Mycobacterium tuberculosis* by automated DNA sequencing. Arch. Pathol. Lab. Med. 119, 131–138. 7848059

[B24] Krüüner A. Yates M. D. Drobniewski F. A. (2006). Evaluation of MGIT 960-based antimicrobial testing and determination of critical concentrations of first- and second-line antimicrobial drugs with drug-resistant clinical strains of *Mycobacterium tuberculosis*. J. Clin. Microbiol. 44, 811–818. 10.1128/JCM.44.3.811-818.2006 16517859 PMC1393078

[B25] Luciani F. Sisson S. A. Jiang H. Francis A. R. Tanaka M. M. (2009). The epidemiological fitness cost of drug resistance in *Mycobacterium tuberculosis*. Proc. Natl. Acad. Sci. U.S.A. 106, 14711–14715. 10.1073/pnas.0902437106 19706556 PMC2732896

[B26] Mahajan R. (2013). Bedaquiline: first FDA-approved tuberculosis drug in 40 years. Int. J. Appl. Basic Med. Res. 3, 1–2. 10.4103/2229-516X.112228 23776831 PMC3678673

[B27] Morens D. M. Folkers G. K. Fauci A. S. (2004). The challenge of emerging and re-emerging infectious diseases. Nature 430, 242–249. 10.1038/nature02759 15241422 PMC7094993

[B28] Murray M. G. Thompson W. F. (1980). Rapid isolation of high molecular weight plant DNA. Nucleic Acids Res. 8, 4321–4325. 10.1093/nar/8.19.4321 7433111 PMC324241

[B29] National Research Council (2010). Guide for the Care and Use of Laboratory Animals, 8th Edn. Washington, DC: The National Academy Press.

[B30] Nouvel L. X. Kassa-Kelembho E. Vultos T. D. Zandanga G. Rauzier J. Lafoz C.. (2006). Multidrug-resistant *Mycobacterium tuberculosis*, Bangui, Central African Republic. Emerg. Infect. Dis. 12, 1454–1456. 10.3201/eid1209.060361 17073103 PMC3298286

[B31] Okamoto S. Tamaru A. Nakajima C. Nishimura K. Tanaka Y. Tokuyama S.. (2007). Loss of a conserved 7-methylguanosine modification in 16S rRNA confers low-level streptomycin resistance in bacteria. Mol. Microbiol. 63, 1096–1106. 10.1111/j.1365-2958.2006.05585.x 17238915

[B32] Piddock L. J. V. (2012). The crisis of no new antibiotics – what is the way forward? Lancet Infect. Dis. 12, 249–253. 10.1016/S1473-3099(11)70316-4 22101066

[B33] Pietersen E. Ignatius E. Streicher E. M. Mastrapa B. Padanilam X. Pooran A.. (2014). Long-term outcomes of patients with extensively drug-resistant tuberculosis in South Africa: a cohort study. Lancet 383, 1230–1239. 10.1016/S0140-6736(13)62675-6 24439237

[B34] Pym A. S. Saint-Joanis B. Cole S. T. (2002). Effect of *katG* mutations on the virulence of *Mycobacterium tuberculosis* and the implication for transmission in humans. Infect. Immun. 70, 4955–4960. 10.1128/IAI.70.9.4955-4960.2002 12183541 PMC128294

[B35] Ramaswamy S. V. Reich R. Dou S. J. Jasperse L. Pan X. Wanger A.. (2003). Single nucleotide polymorphisms in genes associated with isoniazid resistance in *Mycobacterium tuberculosis*. Antimicrob. Agents Chemother. 47, 1241–1250. 10.1128/AAC.47.4.1241-1250.2003 12654653 PMC152487

[B36] Reading C. Cole M. (1977). Clavulanic acid: a beta-lactamase-inhiting beta-lactam from *Streptomyces clavuligerus*. Antimicrob. Agents Chemother. 11, 852–857. 10.1128/AAC.11.5.852 879738 PMC352086

[B37] Rodrigues L. Villellas C. Bailo R. Viveiros M. Aínsa J. A. (2013). Role of the Mmr efflux pump in drug resistance in *Mycobacterium tuberculosis*. Antimicrob. Agents Chemother. 57, 751–757. 10.1128/AAC.01482-12 23165464 PMC3553716

[B38] Ross M. H. Pawlina W. (2006). Histology. Baltimore, MD: Lippincott Williams & Wilkins.

[B39] Sandgren A. Strong M. Muthukrishnan P. Weiner B. K. Church G. M. Murray M. B. (2009). Tuberculosis drug resistance mutation database. PLoS Med. 6:e1000002. 10.1371/journal.pmed.1000002 19209951 PMC2637921

[B40] Scorpio A. Zhang Y. (1996). Mutations in *pncA*, a gene encoding pyrazinamidase/nicotinamidase, cause resistance to the antituberculous drug pyrazinamide in tubercle bacillus. Nat. Med. 2, 662–667. 10.1038/nm0696-662 8640557

[B41] Segal W. Bloch H. (1956). Biochemical differentiation of *Mycobacterium tuberculosis* grown *in vivo* and *in vitro*. J. Bacteriol. 72, 132–141. 13366889 10.1128/jb.72.2.132-141.1956 PMC357869

[B42] Spellberg B. Guidos R. Gilbert D. Bradley J. Boucher H. W. Scheld W. M. Jr.. (2008). Infectious diseases society of America. The epidemic of antibiotic-resistant infections: a call to action for the medical community from the infectious diseases society of America. Clin. Infect. Dis. 46, 155–164. 10.1086/524891 18171244

[B43] Sreevatsan S. Stockbauer K. E. Pan X. Kreiswirth B. N. Moghazeh S. L. Jacobs W. R. Jr.. (1997). Ethambutol resistance in *Mycobacterium tuberculosis*: critical role of *embB* mutations. Antimicrob. Agents Chemother. 41, 1677–1681. 9257740 10.1128/aac.41.8.1677 PMC163984

[B44] Tenover F. C. (2001). Development and spread of bacterial resistance to antimicrobial agents: an overview. Clin. Infect. Dis. 33, S108–S115. 10.1086/321834 11524705

[B45] Torrey H. L. Keren I. Via L. E. Lee J. S. Lewis K. (2016). High persister mutants in *Mycobacterium tuberculosis*. PLoS ONE 11:e0155127. 10.1371/journal.pone.0155127 27176494 PMC4866775

[B46] van den Boogaard J. Kibiki G. S. Kisanga E. R. Boeree M. J. Aarnoutse R. E. (2009). New drugs against tuberculosis: problems, progress, and evaluation of agents in clinical development. Antimicrob. Agents Chemother. 53, 849–862. 10.1128/AAC.00749-08 19075046 PMC2650526

[B47] Yu J. Tran V. Li M. Huang X. Niu C. Wang D.. (2012). Both phthiocerol dimycocerosates and phenolic glycolipids are required for virulence of *Mycobacterium marinum*. Infect. Immun. 80, 1381–1389. 10.1128/IAI.06370-11 22290144 PMC3318414

[B48] Zager E. M. McNerney R. (2008). Mutidrug-resistant tuberculosis. BMC Infect. Dis. 8:10. 10.1186/1471-2334-8-10 18221534 PMC2241599

