# Supplementary material for: Genomic Insight into Mechanisms of Reversion of Antibiotic Resistance in Multidrug Resistant Mycobacterium tuberculosis Induced by a Nanomolecular Iodine-Containing Complex FS-1
Source: Front Cell Infect Microbiol. 2017 May 8;7:151. doi: 10.3389/fcimb.2017.00151 (PMC5420568; doi:10.3389/fcimb.2017.00151)
Supplement: Supplementary file 1 [file Table1.DOCX]

**Supplementary Table 1. Average weight of animals in the different experimental groups.**

| **Groups of infected animals** | **Before infection** | **14 days after infection** | | **30 days of treatment** | | **60 days of treatment** | | **After 30 days of recovery** | |
| --- | --- | --- | --- | --- | --- | --- | --- | --- | --- |
|  | **M ± m** | **M ± m** | **ΔM, %** | **M ± m** | **ΔM, %** | **M ± m** | **ΔM, %** | **M ± m** | **ΔM, %** |
| Group 2, positive control | 492.67 ± 63.02 | 478.82 ± 50.24 | −3.0% | 465.33 ± 43.38 | −5.5% | 462.78 ± 37.59 | −6.0% | All died | |
| Group 3, treated with CAA | 529.40 ± 77.30 | 506.68 ± 61.63 | −4.0% | 546.31 ± 79.22 | +3.0% | 606.20 ± 66.17 | +14.5% | 643.2 ± 94.77* | +21.5% |
| Group 4, treated with CAA + FS-1 (2.5 μg/kg) | 468.96 ± 87.26 | 459.88 ± 65.95 | −2.0% | 525.50 ± 72.26 | +12.0% | 589.60 ± 46.09* | +26.0% | 650.2 ± 57.48* | +39% |
| Group 5, treated with CAA + FS-1 (4.0 μg/kg) | 489.4 ± 37.78 | 475.84 ± 36.07 | −3.0% | 553.44 ± 42.22 | +13.0% | 597.30 ± 45.05* | +22.0% | 626.0 ± 20.63* | +28% |

* р≤0.05 for ΔM in comparison to the weight of animals before infection.
