# Supplementary material for: Genomic Insight into Mechanisms of Reversion of Antibiotic Resistance in Multidrug Resistant Mycobacterium tuberculosis Induced by a Nanomolecular Iodine-Containing Complex FS-1
Source: Front Cell Infect Microbiol. 2017 May 8;7:151. doi: 10.3389/fcimb.2017.00151 (PMC5420568; doi:10.3389/fcimb.2017.00151)
Supplement: Supplementary file 2 [file Table2.DOCX]

**Supplementary Table 2. Blood test results for animals from different experimental groups.**

| **Parameters** | **Negative control** | **14 days after infection** | **Groups of infected animals** | | **14^th^ day of treatment** | **30^th^ day of treatment** | | **45^th^ day of treatment** | **60^th^ day of treatment** | **Recovery, 30^th^ day after treatment** |
| --- | --- | --- | --- | --- | --- | --- | --- | --- | --- | --- |
| WBC (white blood cells), 10^9^/l | 8,74 ± 1,57 | 13,70 ± 1,64 | Group 2, positive control | | 9,97 ± 3,67 | 15,7 ± 1,55 | | 18,43 ± 1,16 | 45,97 ± 10,73 | — |
|  |  |  | Group 3, treated with CAA | | 12,90 ± 1,95 | 15,1 ± 3,59 | | 15,07 ± 3,13 | 19,07 ± 5,94 | 24,10 ± 0,26•• |
|  |  |  | Group 4, treated with CAA + FS-1 (2.5 μg/kg) | | 8,29 ± 1,39 | 9,44 ± 2,10 | | 6,38 ± 0,24••• | 6,75 ± 1,74•• | 16,53±0,80•• |
|  |  |  | Group 5, treated with CAA + FS-1 (4.0 μg/kg) | | 8,73 ± 6,13 | 9,36 ± 2,90 | | 7,15 ± 1,19•• | 9,36 ± 2,90• | 10,41 ± 2,68 |
| LYM (lymphocytes), 10^9^/l | 3,70 ± 1,38 | 8,10 ± 1,53 | Group 2, positive control | | 4,84 ± 2,55 | 6,80 ± 1,86 | | 6,99 ± 1,41 | 36,70 ± 10,97 | — |
|  |  |  | Group 3, treated with CAA | | 6,51 ± 1,97 | 6,84 ± 2,97 | | 5,28 ± 3,33 | 9,13 ± 1,41 | 14,07 ± 3,10• |
|  |  |  | Group 4, treated with CAA + FS-1 (2.5 μg/kg) | | 2,04 ± 2,19 | 4,76 ± 1,84 | | 3,23 ± 0,33 | 2,38 ± 0,43• | 8,16±3,22 |
|  |  |  | Group 5, treated with CAA + FS-1 (4.0 μg/kg) | | 4,67 ± 4,88 | 3,84 ± 1,31 | | 3,78 ± 0,78 | 3,84 ± 1,31• | 3,47 ± 1,19 |
| MID (monocytes),10^9^/l | 2,09 ± 0,98 | 2,02 ± 0,22 | Group 2, positive control | | 0,99 ± 0,85 | 0,79 ± 1,1 | | 0,07 ± 0,04 | 3,25 ± 0,20 | — |
|  |  |  | Group 3, treated with CAA | | 0,28 ± 0,31 | 0,82 ± 1,1 | | 0,95 ± 1,08 | 2,60 ± 0,40 | 2,41 ± 0,37 |
|  |  |  | Group 4, treated with CAA + FS-1 (2.5 μg/kg) | | 0,72 ± 0,91 | 0,21 ± 0,18 | | 0,29 ± 0,14 | 0,09 ± 0,03••• | 2,68 ± 2,27 |
|  |  |  | Group 5, treated with CAA + FS-1 (4.0 μg/kg) | | 3,29 ± 5,03 | 1,24 ± 0,56 | | 0,28 ± 0,03 | 1,24 ± 0,56• | 1,12 ± 0,07 |
| GRA (granulocytes), 10^9^/l | 2,94 ± 1,97 | 3,63 ± 1,00 | Group 2, positive control | | 3,72 ± 1,48 | 8,19 ± 1,40 | | 11,37 ± 0,31 | 6,01 ± 1,36 | - |
|  |  |  | Group 3, treated with CAA | | 6,14 ± 1,61 | 7,45 ± 0,36 | | 8,85 ± 1,65 | 7,35 ± 4,29 | 8,11 ± 0,53 |
|  |  |  | Group 4, treated with CAA + FS-1 (2.5 μg/kg) | | 5,54 ± 2,68 | 4,50 ± 1,25 | | 2,96 ± 0,11••• | 4,28 ± 1,31 | 5,70 ± 3,96 |
|  |  |  | Group 5, treated with CAA + FS-1 (4.0 μg/kg) | | 10,51 ± 13,07 | 4,28 ± 2,26 | | 3,08 ± 1,13•• | 4,28 ± 2,26 | 2,83 ± 1,57 |
| RBC (red blood cells), 10^12^/l | 4,06 ± 0,38 | 6,87 ± 3,15 | Group 2, positive control | | 5,03 ± 0,32 | 5,13 ± 0,05 | | 5,26 ± 0,21 | 6,38 ± 2,33 | — |
|  |  |  | Group 3, treated with CAA | | 4,89 ± 0,32 | 5,02 ± 0,29 | | 5,49 ± 0,10 | 5,22 ± 0,40 | 5,12 ± 0,60 |
|  |  |  | Group 4, treated with CAA + FS-1 (2.5 μg/kg) | | 3,89 ± 0,67 | 4,61 ± 0,47 | | 5,04 ± 0,34 | 5,93±0,47 | 6,12 ± 1,43 |
|  |  |  | Group 5, treated with CAA + FS-1 (4.0 μg/kg) | | 5,07 ± 0,27 | 6,10 ± 0,40 | | 5,11 ± 0,21 | 6,10 ± 0,40 | 4,21 ± 0,14 |
| HGB (hemoglobin), g/l | 108,00 ± 5,29 | 133,33 ± 19,6 | Group 2, positive control | | 126,00 ± 7,00 | | 133,33 ± 2,08 | 129,00 ± 3,46 | 151,67 ± 15,63 | — |
|  |  |  | Group 3, treated with CAA | | 125,00 ± 2,65 | | 126,00 ± 4,36 | 133,00 ± 3,00 | 124,67 ± 6,43 | 122,33 ± 10,02 |
|  |  |  | Group 4, treated with CAA + FS-1 (2.5 μg/kg) | | 124,00 ±9 ,17 | | 119,00 ± 10,54 | 124,00 ± 2,65 | 143,67 ± 7,51 | 103,33 ± 32,87 |
|  |  |  | Group 5, treated with CAA + FS-1 (4.0 μg/kg) | | 126,00 ± 4,36 | | 133,67 ± 8,74 | 129,67 ± 3,79 | 133,67 ± 8,74 | 125,67 ± 3,51 |
| HCT (hematocrit), % | 38,40 ± 2,46 | 54,40 ± 10,66 | Group 2, positive control | | 46,60 ± 3,58 | | 49,00 ± 0,53 | 48,33 ± 1,59 | 64,50 ± 22,82 | — |
|  |  |  | Group 3, treated with CAA | | 45,80 ± 1,32 | | 46,77 ± 2,75 | 49,33 ± 1,43 | 49,53 ± 3,97 | 45,33 ± 3,43 |
|  |  |  | Group 4, treated with CAA + FS-1 (2.5 μg/kg) | | 32,00 ± 6,94 | | 42,93 ± 4,89 | 45,13 ± 1,58 | 55,57 ± 4,29 | 39,80 ± 12,17 |
|  |  |  | Group 5, treated with CAA + FS-1 (4.0 μg/kg) | | 47,37 ± 2,32 | | 60,40 ± 5,71 | 47,00 ± 1,76 | 62,07 ± 3,38 | 40,67 ± 2,70 |
| PLT (platelets), 10^9^/l | 840,67 ± 232,49 | 1182,67 ± 270,3 | Group 2, positive control | | 665,67 ± 258,00 | | 381,67 ± 70,85 | 732,67 ± 366,70 | 1113,00 ± 262,39 | — |
|  |  |  | Group 3, treated with CAA | | 619,67 ± 249,13 | | 960,33 ± 246,11• | 991,00 ± 343,51 | 856,67 ± 120,62 | 1262,33 ± 197,71 |
|  |  |  | Group 4, treated with CAA + FS-1 (2.5 μg/kg) | | 1361,67 ± 227,46 | | 931,33 ± 368,54 | 584,67 ± 103,37 | 859,67 ± 187,78 | 994,00 ± 101,00 |
|  |  |  | Group 5, treated with CAA + FS-1 (4.0 μg/kg) | | 1004,67 ± 191,68 | | 939,33 ± 63,69• | 727,00 ± 31,32 | 939,33 ± 63,69 | 700,00 ± 252,12 |
| ALT (alanine aminotransferase), U/l | 42,00 ± 4,85 | 97,37 ± 8,57** | | Group 2, positive control | 124,57 ± 21,97^*^ | | 86,83 ± 4,14^**^ | 60,00 ± 1,97^*^ | 74,43 ± 8,32^*^ | — |
|  |  |  |  | Group 3, treated with CAA | 49,03 ± 25,92^•^ | | 67,17 ± 9,96 | 87,43 ± 2,14^•••^ | 119,70 ± 9,40 | 61,70 ± 5,67 |
|  |  |  |  | Group 4, treated with CAA + FS-1 (2.5 μg/kg) | 85,53 ± 7,71 | | 62,43 ± 21,76 | 41,90 ± 6,21^•^ | 45,83 ± 1,99^•^ | 61,43 ± 2,24 |
|  |  |  |  | Group 5, treated with CAA + FS-1 (4.0 μg/kg) | 86,53±26,99 | | 77,87±7,91 | 41,40 ± 4,62^•^ | 42,03±5,10^•^ | 62,77±5,40 |
| AST (aspartate aminotransferase), U/l | 178,13 ± 1,14 | 256,27 ± 24,05* | | Group 2, positive control | 264,03±29,11 | | 125,70±28,67 | 101,97±11,59 | 126,60±21,94 | — |
|  |  |  |  | Group 3, treated with CAA | 147,07 ± 43,86 | | 125,47 ± 52,62 | 140,87 ± 34,44 | 158,63±59,07 | 82,44±18,66 |
|  |  |  |  | Group 4, treated with CAA + FS-1 (2.5 μg/kg) | 190,50 ± 43,71 | | 121,23 ± 6,07 | 129,60 ± 60,54 | 142,13 ± 68,15 | 95,47 ± 28,66 |
|  |  |  |  | Group 5, treated with CAA + FS-1 (4.0 μg/kg) | 160,43 ± 37,64 | | 85,97 ± 32,78 | 80,57 ± 21,91 | 83,43 ± 11,30 | 83,20 ± 14,92 |
| Bilirubin total mg/dL | 0,37 ± 0,01 | 0,60 ± 0,08* | | Group 2, positive control | 0,53 ± 0,28 | | 0,35 ± 0,01 | 0,36±0,06 | 0,31 ± 0,13 | — |
|  |  |  |  | Group 3, treated with CAA | 0,46 ± 0,09 | | 0,47 ± 0,10 | 0,46 ± 0,18 | 0,58 ± 0,14 | 0,44 ± 0,09 |
|  |  |  |  | Group 4, treated with CAA + FS-1 (2.5 μg/kg) | 0,67 ± 0,28 | | 0,71 ± 0,42 | 0,53 ± 0,06 | 0,43 ± 0,05 | 0,41 ± 0,20 |
|  |  |  |  | Group 5, treated with CAA + FS-1 (4.0 μg/kg) | 0,42 ± 0,07 | | 0,23 ± 0,02^••^ | 0,43 ± 0,13 | 0,21 ± 0,03 | 0,35 ± 0,07^*^ |
| ALP-AMP (alkaline phosphatase), U/l | 49,70 ± 2,80 | 67,13 ± 19,76 | | Group 2, positive control | 149,43 ± 10,36 | | 65,70 ± 3,90* | 86,07 ± 4,22* | 79,43 ± 25,56,17* | — |
|  |  |  | | Group 3, treated with CAA | 79,77 ± 24,87 | | 39,30 ± 7,34• | 178,13 ± 22,77• | 160,83 ± 7,56• | 64,34 ± 3,67 |
|  |  |  | | Group 4, treated with CAA + FS-1 (2.5 μg/kg) | 130,50 ± 32,95 | | 106,53 ± 49,18 | 98,17 ± 53,87 | 71,67 ± 17,35 | 73,93 ± 66,21 |
|  |  |  | | Group 5, treated with CAA + FS-1 (4.0 μg/kg) | 168,37 ± 18,52 | | 155,23 ± 39,41 | 92,80 ± 87,63 | 142,33 ± 49,10 | 74,00 ± 14,13 |
| Amylase,U/l | 1400,55 ± 11,95 | 1795,89 ± 501,44 | | Group 2, positive control | 1217,51 ± 20,29^**^ | | 1350,54 ± 6,94^**^ | 1292,28 ± 28,34^*^ | 505,21 ± 32,83 | — |
|  |  |  |  | Group 3, treated with CAA | 1454,37 ± 692,59 | | 1297,51 ± 111,08 | 1719,17 ± 234,07 | 1719,17 ± 234,07^•••^ | 1972,35 ± 68,22 |
|  |  |  |  | Group 4, treated with CAA + FS-1 (2.5 μg/kg) | 1199,60 ± 595,57 | | 1169,88 ± 304,54 | 1261,77 ± 79,78 | 1331,50 ± 290,11^•^ | 1244,38 ± 75,62 |
|  |  |  |  | Group 5, treated with CAA + FS-1 (4.0 μg/kg) | 1438,67 ± 51,91^*^ | | 1364,19 ± 102,20 | 1183,94 ± 249,14 | 1283,94 ± 171,59^•^ | 1385,65 ± 32,83 |
| Creatinine, mg/dL | 0,84 ± 0,01 | 0,77 ± 0,21 | | Group 2, positive control | 0,53 ± 0,02^***^ | | 0,53 ± 0,03^***^ | 0,65±0,14^***^ | 0,47±0,03^***^ | — |
|  |  |  |  | Group 3, treated with CAA | 0,65 ± 0,08 | | 0,67 ± 0,07 | 0,57 ± 0,11 | 0,59 ± 0,05 | 0,75 ± 0,07 |
|  |  |  |  | Group 4, treated with CAA + FS-1 (2.5 μg/kg) | 0,50 ± 0,04 | | 0,59 ± 0,07 | 0,57± 0,01 | 0,61± 0,06 | 0,70 ± 0,10 |
|  |  |  |  | Group 5, treated with CAA + FS-1 (4.0 μg/kg) | 0,45 ± 0,05 | | 0,59 ± 0,05 | 0,55 ± 0,12 | 0,58 ± 0,04 | 0,64 ± 0,07 |
| Urea, mg/dL | 109,03 ± 0,67 | 99,77 ± 34,13 | | Group 2, positive control | 23,87 ± 5,78^***^ | | 39,63 ± 1,39^***^ | 38,57±3,82^***^ | 32,93±1,21^***^ | — |
|  |  |  |  | Group 3, treated with CAA | 39,10 ± 10,34 | | 37,93 ± 16,82 | 20,67 ± 8,29 | 50,43 ± 15,63 | 63,64 ± 5,93 |
|  |  |  |  | Group 4, treated with CAA + FS-1 (2.5 μg/kg) | 44,80 ± 1,57^•^ | | 53,97 ± 9,20 | 51,33 ± 1,40^•^ | 46,80 ± 6,42 | 51,60 ± 8,85 |
|  |  |  |  | Group 5, treated with CAA + FS-1 (4.0 μg/kg) | 47,63 ± 4,77^•^ | | 86,30 ± 14,88^•^ | 52,87 ± 9,97 | 47,50 ± 8,85 | 55,03 ± 4,82 |
| Glucose, mmol/l | 7,27 ± 0,11 | 7,62 ± 1,98 | | Group 2, positive control | 7,75 ± 0,17 | | 8,01 ± 0,53 | 8,26±1,06 | 8,80±0,49*• | — |
|  |  |  | | Group 3, treated with CAA | 8,35±1,36 | | 6,13±0,74 | 6,71±0,66 | 7,81±1,80 | 6,37±0,83 |
|  |  |  | | Group 4, treated with CAA + FS-1 (2.5 μg/kg) | 7,77±0,35 | | 7,30±1,25 | 6,50±1,98 | 6,77±0,43 | 9,64±2,32 |
|  |  |  | | Group 5, treated with CAA + FS-1 (4.0 μg/kg) | 6,42±0,33• | | 5,26±0,39• | 6,86±0,44 | 11,98±2,75 | 9,88±2,68 |
| Protein total, g/l | 57,63 ± 0,49 | 50,40 ± 2,72 | | Group 2, positive control | 48,40±0,36*** | | 54,20±0,26** | 64,50±4,94 | 55,50±5,13 | — |
|  |  |  | | Group 3, treated with CAA | 48,27±3,76 | | 54,27±5,82 | 53,07±1,07 | 56,00±2,19 | 57,84±1,61 |
|  |  |  | | Group 4, treated with CAA + FS-1 (2.5 μg/kg) | 46,60±1,74 | | 51,60±1,15 | 56,83±4,12• | 54,10±5,12 | 57,73±5,74 |
|  |  |  | | Group 5, treated with CAA + FS-1 (4.0 μg/kg) | 49,63 ± 0,90 | | 49,93 ± 1,00• | 52,13 ± 3,07 | 52,87 ± 5,12 | 57,40 ± 3,50 |
| Albumin, g/l | 22,27 ± 0,06 | 16,43 ± 0,51*** | | Group 2, positive control | 19,27 ± 0,47** | | 17,60 ± 0,35*** | 21,63 ± 1,77 | 20,47 ± 0,96 | — |
|  |  |  | | Group 3, treated with CAA | 16,07 ± 1,88 | | 19,33 ± 0,78 | 17,97 ± 1,50 | 21,20 ± 0,62 | 18,60 ± 1,79 |
|  |  |  | | Group 4, treated with CAA + FS-1 (2.5 μg/kg) | 17,47 ± 1,00 | | 20,77 ± 0,31•• | 19,20 ± 0,35 | 22,23 ± 1,25 | 18,07 ± 2,40 |
|  |  |  | | Group 5, treated with CAA + FS-1 (4.0 μg/kg) | 20,07 ± 0,12 | | 18,97 ± 0,86 | 18,40 ± 0,40 | 20,97± 0,70 | 22,80 ± 1,80 |
| Triglycerides, mg/dL | 223,90 ± 29,08 | 172,04±27,01^***^ | | Group 2, positive control | 40,44 ± 11,39^**^ | | 62,96 ± 0,33^*^ | 99,88 ± 22,89^*^ | 96,41 ± 33,39^*^ | — |
|  |  |  |  | Group 3, treated with CAA | 134,60 ± 65,37 | | 99,03 ± 46,81 | 126,30 ± 37,47 | 121,09 ± 15,48 | 63,12 ± 42,80 |
|  |  |  |  | Group 4, treated with CAA + FS-1 (2.5 μg/kg) | 65,65 ± 1,60 | | 138,29 ± 47,93 | 46,94 ± 2,81 | 111,54±27,45 | 79,63 ± 21,19 |
|  |  |  |  | Group 5, treated with CAA + FS-1 (4.0 μg/kg) | 88,08 ± 14,76 | | 121,29 ± 20,6•4 | 83,23 ± 23,12 | 84,31 ± 6,68 | 90,48 ± 27,08 |
| Cholesterol, mg/dL | 97,93 ± 0,51 | 42,40 ± 15,82^**^ | | Group 2, positive control | 39,00 ± 3,30^***^ | | 30,93 ± 0,61^***^ | 43,37 ± 6,76^***^ | 53,67 ± 1,40^***^ | — |
|  |  |  |  | Group 3, treated with CAA | 32,53 ± 8,93 | | 46,53 ± 24,28 | 60,13 ± 5,08 | 37,73 ± 8,47 | 42,36 ± 9,38 |
|  |  |  |  | Group 4, treated with CAA + FS-1 (2.5 μg/kg) | 47,77 ± 6,93 | | 47,00 ± 8,18 | 51,40 ± 7,63 | 46,57 ± 7,52 | 58,63 ± 7,35 |
|  |  |  |  | Group 5, treated with CAA + FS-1 (4.0 μg/kg) | 43,97 ± 7,73 | | 55,10 ± 8,00^•^ | 53,13 ± 5,48 | 48,60 ± 5,66 | 53,03 ± 5,34 |
| Fe, mg/dL | 54,60 ± 0,26 | 28,50 ± 11,81 | | Group 2, positive control | 37,13 ± 9,70 | | 43,73 ± 0,59 | 56,43 ± 6,15 | 40,70 ± 9,81 | — |
|  |  |  |  | Group 3, treated with CAA | 33,63 ± 23,62 | | 54,60 ± 5,96 | 37,67± 14,76 | 54,00 ± 3,93 | 48,68 ± 4,12 |
|  |  |  |  | Group 4, treated with CAA + FS-1 (2.5 μg/kg) | 46,00 ± 5,76 | | 59,77 ± 3,43^•^ | 52,17 ± 7,08 | 56,50 ± 3,97 | 49,17 ± 0,50 |
|  |  |  |  | Group 5, treated with CAA + FS-1 (4.0 μg/kg) | 55,90 ± 7,17 | | 43,77 ± 12,29 | 49,23 ± 1,97 | 50,30 ± 3,52 | 52,70 ± 3,56 |
| Ca, mg/dL | 13,08 ± 0,55 | 9,03 ± 0,21 | | Group 2, positive control | 10,77 ± 0,45^*^ | | 10,51 ± 0,55 | 9,99 ± 0,65^*^ | 10,98 ± 0,11 | — |
|  |  |  |  | Group 3, treated with CAA | 10,57 ± 0,66 | | 10,47 ± 0,45 | 11,33 ± 1,56 | 9,41 ± 0,09^•^ | 9,30 ± 0,58 |
|  |  |  |  | Group 4, treated with CAA + FS-1 (2.5 μg/kg) | 10,99 ± 0,33 | | 10,53 ± 0,35 | 12,81 ± 0,12 | 13,43 ± 0,63^•^ | 11,17 ± 0,70 |
|  |  |  |  | Group 5, treated with CAA + FS-1 (4.0 μg/kg) | 8,41 ± 0,90 | | 10,07 ± 0,67 | 10,34 ± 0,78 | 13,17 ± 0,73^•^ | 11,86 ± 0,06 |

• – Р≤0,05; •• – Р≤0,01 and ••• – Р≤0,001 compared to the negative control.
