# Supplementary material for: Genomic Insight into Mechanisms of Reversion of Antibiotic Resistance in Multidrug Resistant Mycobacterium tuberculosis Induced by a Nanomolecular Iodine-Containing Complex FS-1
Source: Front Cell Infect Microbiol. 2017 May 8;7:151. doi: 10.3389/fcimb.2017.00151 (PMC5420568; doi:10.3389/fcimb.2017.00151)
Supplement: Supplementary file 3 [file Image1.PDF]

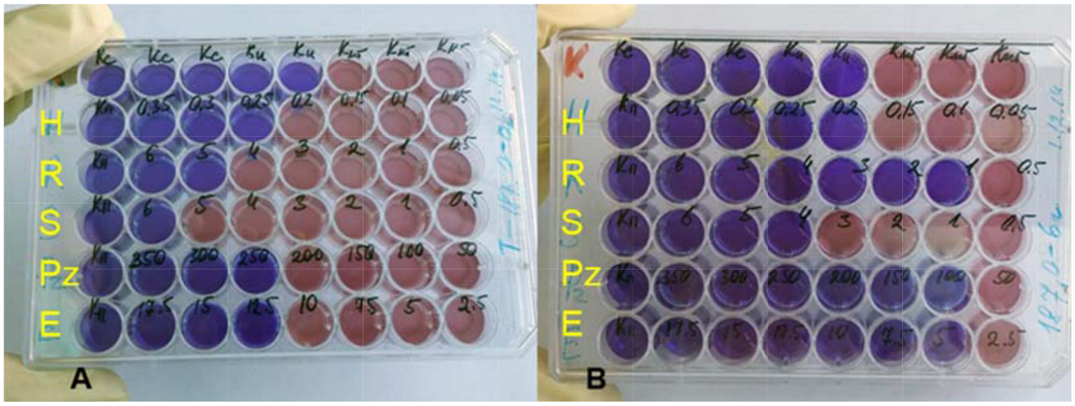

Supplementary Figure 1. Antibiotic resistance reversion in an *in vitro* experiment after 60 days of cultivation of the extensively drug resistan (XDR) strain *M. tuberculosis* SCAID 187.0 in the medium with a sublethal concentration of 12 µg/ml of FS-1. Antibiotics are abbreviated: H - isoniazide; R - rifampicin; S - streptomycin; Pz - pyrazinamide; E - ethionamide. Top left 5 wells are negative control of contamination. Concentrations of the corresponding antibiotics in every well are given in µg/ml. Pink colour of the medium indicates the bacterial growth; blue - absence of growth. **A:** sensitivity to the antibiotics after 60 days of cultivation on the medium containing 12 µg/ml of FS-1. **B:** sensitivity to the antibiotics after 60 days of cultivation on the medium without FS-1.
