# Supplementary material for: Genomic Insight into Mechanisms of Reversion of Antibiotic Resistance in Multidrug Resistant Mycobacterium tuberculosis Induced by a Nanomolecular Iodine-Containing Complex FS-1
Source: Front Cell Infect Microbiol. 2017 May 8;7:151. doi: 10.3389/fcimb.2017.00151 (PMC5420568; doi:10.3389/fcimb.2017.00151)
Supplement: Supplementary file 4 [file Image2.PDF]

Group 2, positive control (infected animals)	Group 3, animals treated with CAA	Group 5, animals treated with CAA + FS-1 (4.0 mg/kg)
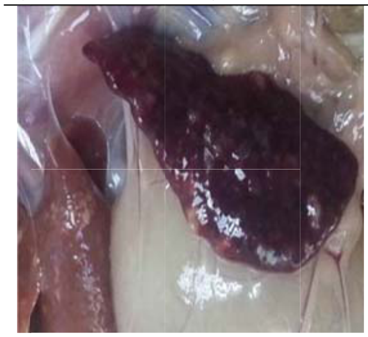	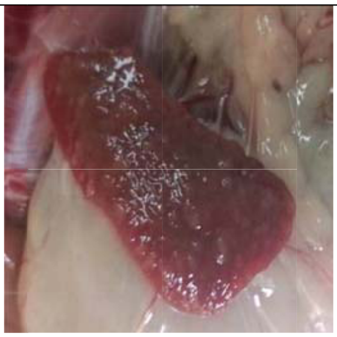	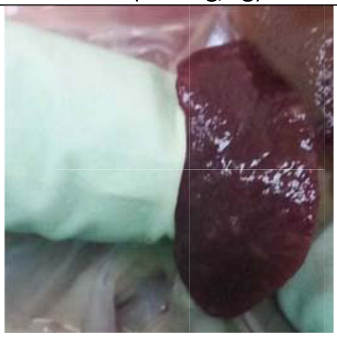
	**Spleen**	
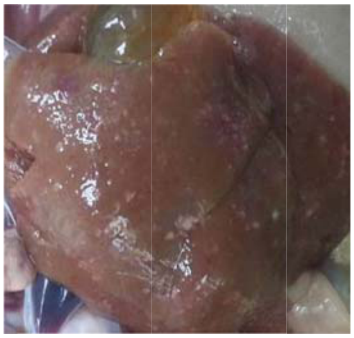	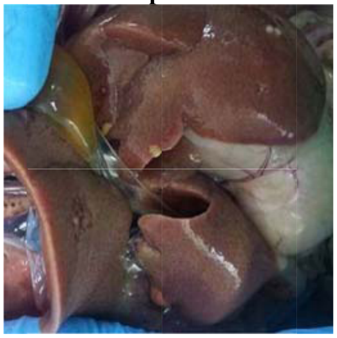	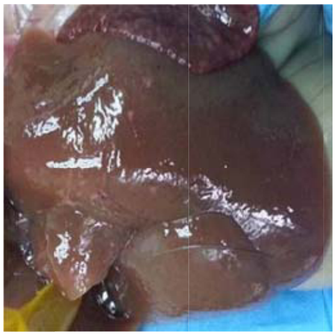
	**Liver**	
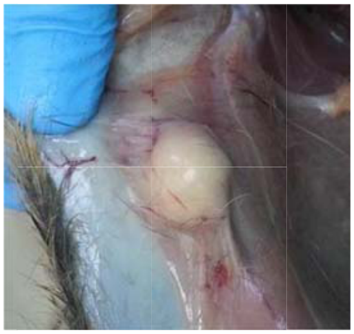	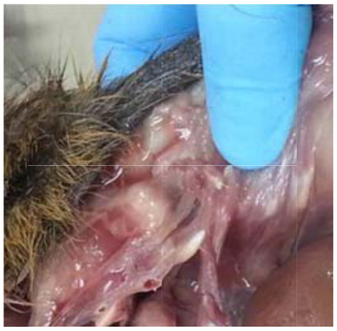	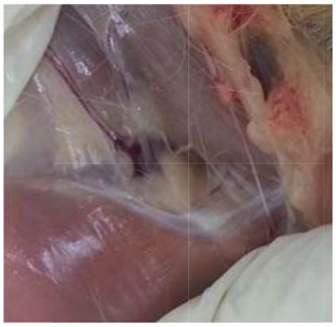
	**Lymph nodes**	

A: Animals on the 45th day of treatment.

Group 2, positive control (infected animals)	Group 3, animals treated with CAA	Group 5, animals treated with CAA + FS-1 (4.0 mg/kg)

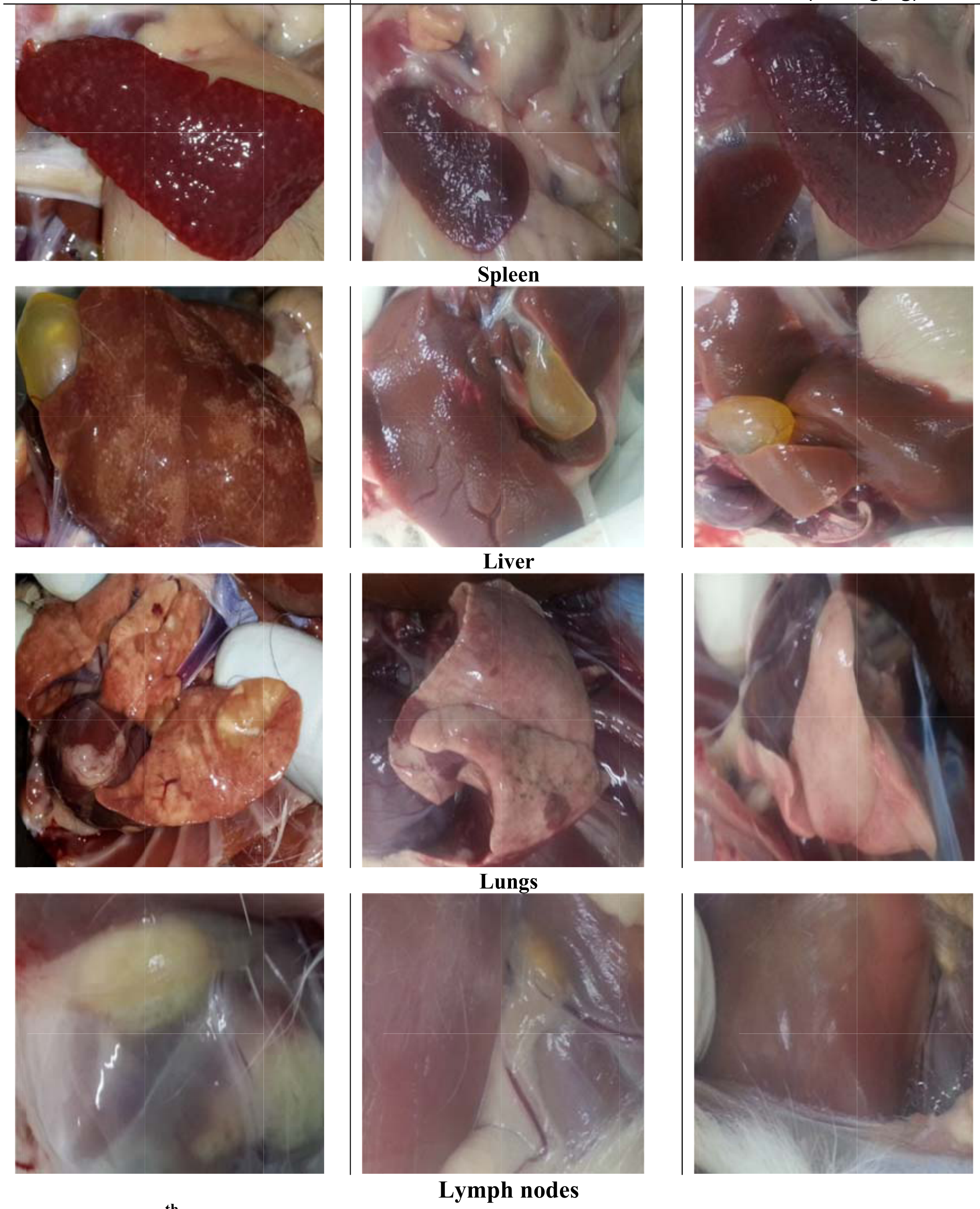

B: Animals on the 60th day of treatment.

Supplementary Figure 2. Photos of visera of infected animals of the positive contol and the animals under different treatment regiments.
