# Supplementary material for: Genomic Insight into Mechanisms of Reversion of Antibiotic Resistance in Multidrug Resistant Mycobacterium tuberculosis Induced by a Nanomolecular Iodine-Containing Complex FS-1
Source: Front Cell Infect Microbiol. 2017 May 8;7:151. doi: 10.3389/fcimb.2017.00151 (PMC5420568; doi:10.3389/fcimb.2017.00151)
Supplement: Supplementary file 5 [file Image3.PDF]

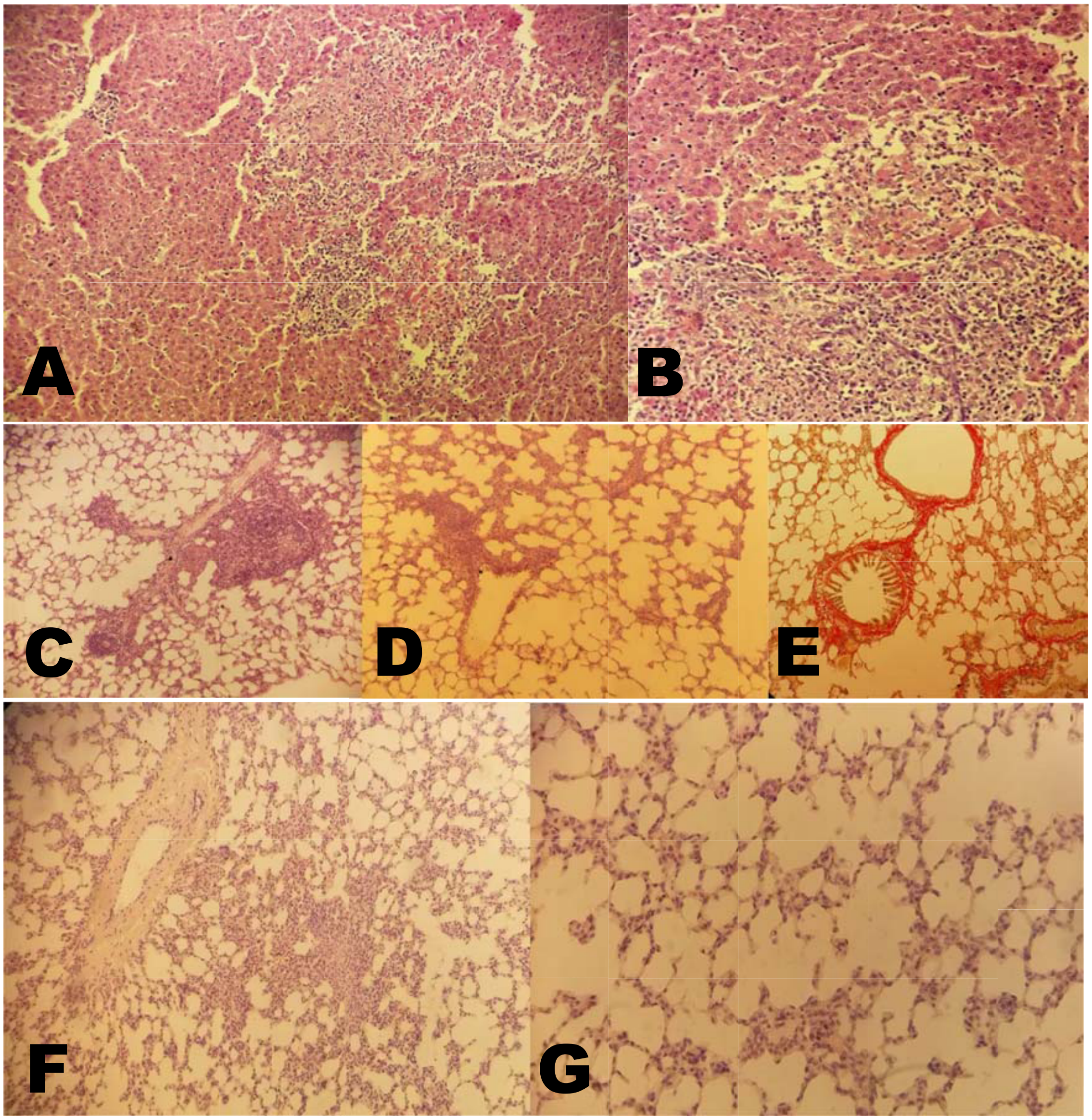

Supplementary Figure 3. Photomicrography of sections of viscera of infected animals from the positive control group (untreated) animals (sections **A-E**); and from the animals treated with CAA + FS-1 (sections **F-G**). **A and B**: liver tissues, the 21st day after infection, stained with hematoxylin and eosin, magnitude ×20 and ×40, respectively; **C and D**: lung tissues, the 35st day after infection, stained with hematoxylin and eosin, magnitude ×20; **E**: lung tissues, the 35st day after infection, Van Gieson's stain, magnitude ×20; **F and G**: lung tissues, the 60st day of the treatment with CAA + FS-1 (2.5 and 4.0 mg/kg, respectively), stained with hematoxylin and eosin, magnitude ×10. Microscopy of the lung tissue sections revealed inflammatory infiltrations in all layers of bronchioles. Alveolar walls were thickened and congested, or in others cases – thinned. Secondary and tertiary bronchi were swollen with desquamated epithelium. Alveolar duct contained exudate. Other alveoli were swollen with gas. Peripheral location of nuclei was observed in some alveolar cells. The pathological state of lungs was diagnosed as bronchopneumonia with perifocal emphysema. Microscopy of the histological sections prepared from the sacrificed infected animals prior to the treatment showed a serious disruption of the cytoarchitecture of the spleen with multiple Langhans giant cells. In liver sections taken from tubercles, caseous necrosis was observed in the form of unstructured pale-pink agglomerations of cell particles surrounded by infiltration zones with epithelioid cells, lymphocytes and Langhans giant cells. Hepatic cells around the tubercles were hypereosinophilic. The microscopy also revealed widening of the perisinusoidal Disse space in the liver. Proliferative activity of macrophages and parenchymal proteinosis of hepatocytes with fat inclusion bodies and doubled nuclei were observed. After 60 days of the combined treatment the hepatic tissue and pulmonary parenchyma of the animals was close to the normal state with good lung airiness. Porous perivascular infiltrates and lymphocyte infiltrates into interalveolar walls were observed in lungs only in a few animals.
